# Effect of Shikimic Acid on Oxidation of Myofibrillar Protein of Duck Meat During Heat Treatment

**DOI:** 10.3390/foods13203338

**Published:** 2024-10-21

**Authors:** Yue Niu, Yingrui Zhang, Yuwei Wang, Wenjie He, Wei Xu, Danjun Guo, Hongxun Wang, Yang Yi, Guowei Tan

**Affiliations:** 1 College of Food Science and Engineering, Wuhan Polytechnic University, Wuhan 430023, China; 17871151560@163.com (Y.N.); 16637687827@163.com (Y.Z.); 13669077749@163.com (Y.W.); hhh19970401@163.com (W.H.); missguodj@163.com (D.G.); yiy86@whpu.edu.cn (Y.Y.); tgw19872060272@163.com (G.T.); 2 Hubei Key Laboratory for Processing and Transformation of Agricultural Products, Wuhan 430023, China; wanghongxunhust@163.com; 3 College of Life Science and Technology, Wuhan Polytechnic University, Wuhan 430023, China

**Keywords:** shikimic acid, duck meat, myofibrillar protein, antioxidation, protein structure

## Abstract

The myofibrillar protein (MP) of duck meat is prone to excessive oxidation during thermal processing, resulting in a decline in its overall quality. In this paper, the effect of shikimic acid on the oxidative structure of duck muscle fibrin was studied. The findings showed that, at a mass ratio of 1:50,000 (g/g) between shikimic acid and MP, the carbonyl content of MP was reduced by 74.20%, while the sulfhydryl content was increased by 73.56%. MP demonstrated the highest denaturation temperature, whereas its thermal absorption was the lowest. The percentage of α-helixes and β-sheets increased by 16.72% and 24.74%, respectively, while the percentage of irregular structures decreased by 56.23%. In addition, the surface hydrophobicity index of MP exhibited a significant decrease (*p* < 0.05), while there was a significant increase in its free radical-scavenging ability (*p* < 0.05). Molecular fluorescence spectrum analysis showed that shikimic acid could bind to MP, altering the internal environment of MP and enhancing its thermal stability. FTIR analysis showed that shikimic acid could enhance the distribution of protein particle sizes by reducing irregular structures, the proportion of β-rotation, and the degree of protein aggregation. It is hoped that this research can offer scientific support for improving meat processing technology.

## 1. Introduction

Duck meat is a high protein, low fat, and low cholesterol food [1]. Heat treatment is a common and important method of processing duck meat to improve food safety, texture, and flavour and increase its storage life, providing a better eating experience. Some studies have found that high temperatures destroy the nutrients in duck meat, such as vitamins and antioxidant substances, reducing its nutritional value [2,3]. It has also been shown that excessive heat treatment leads to changes in the texture of duck meat, significantly reducing its tenderness and flavour [4]. These specific changes were closely related to myofibrillar protein in duck meat.

Myofibrillar protein plays a significant role in meat composition, constituting approximately 50–55% of the overall muscle protein content. Studies have shown that myofibrillar proteins make the connections between muscle fibres tighter. It increases tensile strength and adhesion of muscle fibres, which affect the tenderness of the muscles [5,6]. Myofibrillar proteins are susceptible to oxidation during heat treatment. The oxidation of proteins has been reported to reduce the water-holding capacity and texture of processed meat products, which affects their tenderness and juiciness [7,8]. Oxidation exposes the sulfhydryl groups of myofibrillar proteins, which, in turn, cross-link with other proteins, leading to protein aggregation [9]. Oxidation also affects the conformation and function of myofibrillar proteins. Myofibrillar proteins with a higher degree of oxidation exhibit stronger hydrophobicity, resulting in enhanced protein interactions and further decreasing tenderness [10]. Therefore, taking into account consumers’ demand for products with good flavour and high nutritional value, it is necessary to solve the problem of adverse changes in meat during hot processing.

It is widely accepted that the addition of appropriate antioxidants can reduce the extent of myofibrillar protein oxidation and maintain meat product tenderness [11,12,13]. As the demand for healthy food and natural ingredients increases, the research and application of natural antioxidants are gradually gaining attention. Among them are spices commonly used in culinary processes. These can bring a unique flavour to the taste buds and also have antioxidant properties. Studies have shown that flavonoids in spices can improve the sensory quality of meat products by inhibiting oxidative damage to myofibrillar protein [14]. Star anise (*Illicium verum*) is a spice widely used in meat products, and its main active component is shikimic acid [15]. Previous laboratory studies have found that Star anise extract can improve the tenderness of meat products. When duck meat was braised for 45 min, the shear force in the Star anise extract group was reduced by 40.87%, and the myofibrillar fracture index increased by 16.13% compared with the blank group. The content of shikimic acid in Star anise can reach 6.28%~11.57% [16]. The reason why the extract of Star anise can improve the tenderness of duck meat may be that shikimic acid can change the structure of duck myofibrillar protein (MP) and improve its processing quality. In this study, spectral analysis and other methods were used to determine the interaction between shikimic acid and duck myofibrillar proteins. The effect of shikimic acid on the structure of myofibrillar protein during heat treatment and its mechanism were investigated. The purpose of this study was to provide a theoretical basis for the application of shikimic acid in meat processing.

## 2. Materials and Methods

### 2.1. Materials and Reagents

Duck meat was purchased from Guangxi Dongxin Duck Co., Ltd. (Liuzhou, China). Shikimic acid was purchased from Guilin Rhein Biotechnology Co., Ltd. (Guilin, China). Golden Arowana soybean oil was purchased from Yihai Kerry Grain and Oil Industry Co., Ltd. (Wuhan, China). Spices purchased at Zhongbai Supermarket (Wuhan, China). Magnesium chloride, potassium chloride, disodium glycolate tetraacetic acid, ethanol, trifluoroacetic acid, Phenylmethanesulfonyl fluoride, formic acid, and β-mercaptoethanol were manufactured by Sinopath Chemical Reagents Co., Ltd. (Shanghai, China). All chemicals and reagents were analytical grade.

### 2.2. Methods

#### 2.2.1. Preparation of Duck Meat Myofibrillar Protein

Duck myofibrillar protein was prepared using a modified version of the previously reported method [17]. We washed the fresh duck thoroughly under water, wiped the water off the surface of the duck with absorbent paper, and, finally, cut it into appropriately sized pieces. A 2 g sample of the duck meat was taken and added to 20 mL buffer (150 mmol/L sodium chloride, 25 mmol/L potassium chloride, 3 mmol/L magnesium chloride, 4 mmol/L disodium ethylenediamine tetraacetate, and 1 mmol/L protease inhibitor, pH 6.50). It was homogenised for 30 s at 10,000 rpm and centrifuged for 30 min. The sample containing the myofibrillar protein precipitate was rinsed three times. The aforementioned procedures were carried out at 4 °C. The concentration of the protein mass was determined using the biuret assay, and the isolated protein was kept at 4 °C and utilised within 12 h. The precipitated MP was resuspended in a phosphate buffer, and the concentration of the resulting solution was set to 1 mg/mL. After dissolving, it was put into a polyethylene bag, sealed by vacuum (100 mL/bag), and heated at a constant vacuum temperature of 70 °C for 8 h.

#### 2.2.2. Sample Preparation

According to the laboratory treatment method, MP was placed in a water bath at 100 °C for 10 min, and different amounts of shikimic acid were added to it. The mass-specific concentrations of shikimic acid and MP were 1:50,000, 1:25,000, 1:12,500, and 1:6250 (g/g). They were placed in a 100 °C water bath for an additional 30 min. The blank group consisted of an MP solution without shikimic acid. Samples were taken from the final prepared mixed solution as well as the blank group sample solution in each water bath for a duration of 10 min.

#### 2.2.3. Determination of Carbonyl Content

A method referred to by Rodriguez-Carpena et al. was slightly modified to determine the carbonyl content [18]. A standard curve was constructed using bovine serum protein, and absorbance readings were taken at 280 nm. Additionally, the absorbance of the treated sample was quantified at a wavelength of 370 nm. The molar absorption coefficient of 22,000 L/mol.cm was used to determine the content of carbonyl groups, and Vitamin C (VC) served as a positive control.

#### 2.2.4. Determination of Free Sulfhydryl Content

The free sulfhydryl content was determined using the method developed by Zakrys-Waliwander et al. [19]. The absorbance at 412 nm was measured after 2 mL of sample solution was added to Tris buffer (4 mL, pH = 8) at room temperature for 30 min. The absorption coefficient of the sulfhydryl molecule was 13,600 L/mol.cm, and vitamin C (VC) served as a positive control.

#### 2.2.5. Hydroxyl Radical-Scavenging Activity

A method by Shen et al. was referenced and slightly modified to determine the capacity to neutralise hydroxyl radicals [20]. Sequentially, 1 mL of the sample solution, salicylic acid–ethanol solution (6 mmol/L), ferrous sulfate solution (6 mmol/L), and hydrogen peroxide solution (6 mmol/L) were added to a test tube and mixed well. The absorbance was measured at 510 nm after being held in a water bath maintained at 37 °C for a duration of 30 min. The rate of hydroxyl radical scavenging was calculated using the following formula:

Hydroxyl radical-scavenging activity (%) = [1 − (A_1_ − A_2_) A_0_] × 100% (1)

A_0_—Distilled water instead of blank group and experimental group sample solution absorbance value;
A_1_—Absorbance at 510 nm;
A_2_—The absorbance value of anhydrous ethanol instead of salicylic acid–ethanol solution.

#### 2.2.6. DPPH Radical-Scavenging Activity

The total free radical-scavenging activity of DPPH was determined using the previously reported method with some modifications [21]. A total of 2 mL of the sample solution was thoroughly mixed with 2 mL of a 0.2 mM/L DPPH solution and left to stand away from light (30 min). The absorbance at 517 nm was measured three times for each sample, using methanol as a blank control. The absorption value of 2 mL water and 2 mL DPPH was measured as A_0_. The absorption value of the sample solution (2 mL) mixed with 2 mL DPPH was denoted as A_i_. The absorption value of the sample solution (2 mL) mixed with 2 mL methanol was denoted as A_j_. DPPH free radical clearance was calculated according to the following formula:

DPPH scavenging activity (%) = [1 − (A_i_ − A_j_) A_0_] × 100% (2)

#### 2.2.7. Surface Hydrophobicity

The molecular fluorescence was determined according to Turgut et al. [22]. A mixture of shikimic acid and myofibrillar proteins was prepared in a water bath for 15 min, with ratios of 1:50,000, 1:25,000, 1:12,500, and 1:6500 (g/g). The water bath was set at 90 °C for 30 min, and 2 mL of the final mixture and the blank group were taken for fluorescence scanning every 10 min during heating. Fluorescence scanning conditions included a fixed excitation wavelength of 296 nm, an emission wavelength range of 270–350 nm, an excitation and emission slit width of 2.5 nm, and a scanning rate of 12,000 nm/min.

#### 2.2.8. Molecular Fluorescence Assay

Surface hydrophobicity was determined using a previously established method with some modifications [23]. The compound 1-Benzoic acid-8-sulfonic acid was used as a fluorescent probe, and 2 mL of the sample solution was added to the phosphate buffer. The fluorescence intensity at 470 nm was used to plot the protein concentration, and the gradient of the line corresponded to the protein’s degree of surface hydrophobicity.

#### 2.2.9. Particle Size Determination

The method of Huang was referred to and slightly modified for determining the particle size [24]. We measured 1.5 mL of the sample solution and centrifuged it at a speed of 10,000 rpm for 10 min. The particle size distribution of myofibrillar protein in the sample solution was determined using a nanoparticle size analyser (Malvern Instrument Company, Worcester, UK). The temperature measured during the analysis was 25 °C.

#### 2.2.10. DSC Analysis

DSC analyses were performed according to the method of Kocher et al. [25] with minor modifications. A mixture of shikimic acid and myofibrillar proteins was prepared in a water bath for 15 min, with ratios of 1:50,000, 1:25,000, 1:12,500, and 1:6500 (g/g). The samples from each group were analysed using DSC. Hesheng Instrument Technology Company (Shanghai, China) was used.

#### 2.2.11. Fourier Transform Infrared Absorption Spectroscopy Analysis

The approach employed by Nguyen et al. [26] was used and slightly modified in order to perform Fourier transform infrared absorption spectroscopy. A mixture of shikimic acid and myofibrillar proteins was prepared in a water bath for 15 min, with ratios of 1:50,000, 1:25,000, 1:12,500, and 1:6500 (g/g). The water bath was set at 90 °C for 30 min, and 2 mL of the final mixture and the blank group were taken for infrared spectroscopy analysis every 10 min during the water bath. Infrared spectrometry parameters were as follows: the wavelength range was 500–4500 cm^−1^, and the signal acquisition mode was Attenuated Total Reflection (ATR) mode.

### 2.3. Statistical Analysis

Microsoft Excel 2016 and Origin 2018 were used for data analysis and plotting purposes. The variance method in SPSS 19.0 was used to analyse all data. Univariate analysis of variance (ANOVA) and Duncan test were used to analyse the significant differences between the mean values (*p* < 0.05). All data were independently tested for three replicates and expressed as mean ± standard deviation.

## 3. Results and Discussion

### 3.1. Hydroxyl Radical- and DPPH Radical-Scavenging Rate

**Figure 1:**
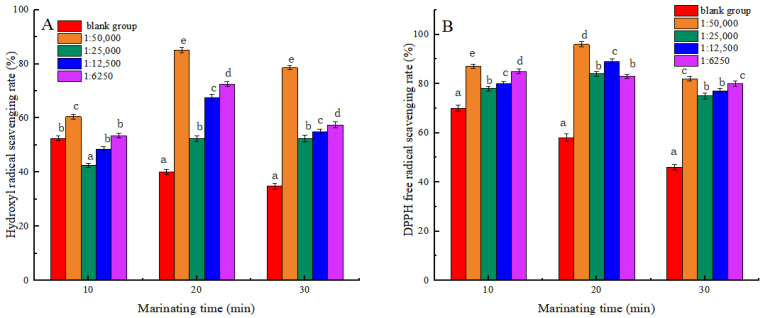
Effects of Shikimic acid on the scavenging rate of myofibrillar protein hydroxyl radicals (**A**) and the scavenging rate of myofibrillar protein DPPH active free radicals (**B**). Note: different letters in the figure indicate significant differences (*p* < 0.05).

Figure 1 shows that the scavenging rates of Hydroxyl radicals and DPPH radicals in the blank group both decreased during heating. The findings indicated that high temperatures could induce the formation of Hydroxyl and DPPH radicals within meat products. These radicals were highly active, which would lead to oxidative damage of proteins and further degradation of proteins [27]. At 20 min and 30 min of heating, the scavenging activities of Hydroxyl and DPPH radicals were significantly increased in all experimental groups compared to the blank group (*p* < 0.05). The results showed that shikimic acid can effectively enhance the scavenging ability of MP against Hydroxyl and DPPH radicals during heat treatment, thus preventing further oxidation of proteins. Some studies have found that plant bioactive compounds can enhance the clearance activity of reactive oxygen species in meat during heating processes, thereby inhibiting the degradation of proteins [28]. At the same heat treatment time, the hydroxyl radical-scavenging capacity was significantly higher in the 1:50,000 group than in other experimental groups (*p* < 0.05). The results may be due to the excess of shikimic acid forming complexes or condensates that prevent free radicals from binding to them, resulting in decreased free radical clearance.

### 3.2. Carbonyl and Free Sulfhydryl Groups

**Figure 2:**
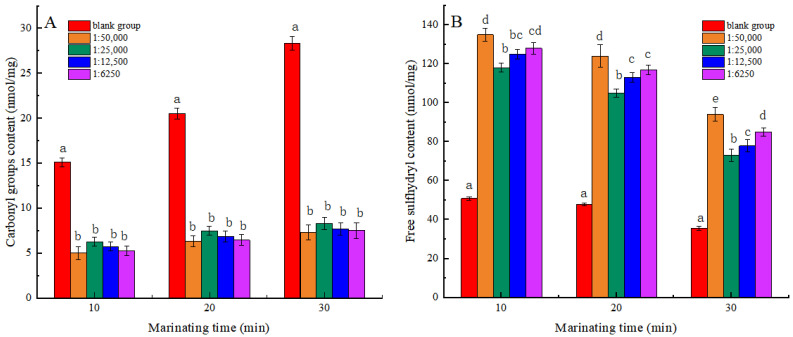
Effects of Shikimic acid on the carbonyl content of myofibrillar protein (**A**) and the free sulfhydryl content of myofibrillar protein (**B**). Note: different letters in the figure indicate significant differences (*p* < 0.05).

Measuring the content of carbonyl groups and free sulfhydryl groups allows for a comprehensive assessment of protein oxidative status [29]. As seen in Figure 2, the carbonyl content of MP tended to increase, while the sulfhydryl content exhibited a downward trend in all groups with prolonged heat treatment duration. The results showed that the oxidation degree of MP increased with the extension of heat treatment time. The carbonylation of amino acid side chains in protein increased the content of carbonyl groups at high temperatures. The free sulfhydryl groups between the polypeptide chains reacted to form disulfide bonds, resulting in a decrease in the free sulfhydryl group content [30]. At the same heat treatment time, a noticeable reduction in carbonyl content and a considerable elevation in free sulfhydryl content were observed among the experimental groups relative to the blank group (*p* < 0.05). The findings demonstrated that shikimic acid significantly mitigated the oxidative state of myofibrillar proteins. This finding was consistent with the outcomes observed in the aforementioned study regarding the activity of scavenging free radicals. When the mass ratio of shikimic acid to myofibrillar protein was 1:50,000 (g/g), and the heat treatment was for 30 min, the carbonyl content of myofibrillar protein reduced by 74.20%, while the sulfhydryl content increased by 73.56% compared to the blank group. The findings indicated that the degree of oxidation of MP was the lowest under these conditions. However, the elevated levels of shikimic acid may disrupt the structure of MP and further increase the degree of protein oxidation.

### 3.3. DSC Analysis

**Figure 3:**
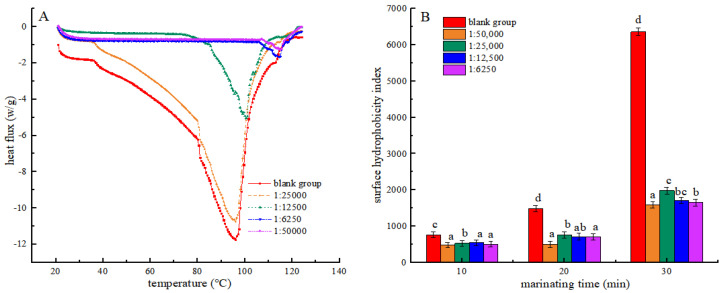
Effects of the Shikimic acid on the thermal denaturation of myofibrillar protein (**A**) and effects of Shikimic acid on the surface hydrophobicity of myofibrillar protein (**B**). Note: different letters in the figure indicate significant differences (*p* < 0.05).

Differential Scanning Calorimetry (DSC) is a thermal analysis technique that serves as an important indicator for evaluating the structural changes of proteins [31]. It was used in this experiment for measuring the energy changes during protein denaturation. As shown in Table 1 and Figure 3A, the denaturation temperature and initial denaturation temperature exhibited an increase across all treatment groups compared to the blank group. This could be due to the interaction between shikimic acid and molecules in myofibrillar protein, which enhances the stability of its internal structure, allowing it to withstand higher temperatures. This was similar to the results of Xu et al. [32]. At a mass ratio of 1:50,000 (g/g) between shikimic acid and protein, the myofibrillar protein exhibited the highest denaturation temperature of 107.35 °C, an initial denaturation temperature of 115.68 °C, and a thermal absorption value as low as 118.42 J·g^−1^. The findings showed that the thermal degeneration of myofibrillar protein was minimal under these conditions. During heat treatment, the incorporation of shikimic acid was found to reduce thermal absorption by myofibrillar protein and mitigate the extent of chemical bond disruption in myofibrillar protein. At the same time, the thermal stability of myofibrillar protein was enhanced to increase its denaturation temperature. However, the high shikimic acid content may disrupt the internal chemical bonds of myofibrillar protein, thereby reducing its thermal stability.

### 3.4. Surface Hydrophobicity

The hydrophobicity of the myofibrillar protein surface reflects both the thermal-induced degradation of its hydrophobic region and the extent of protein cross-linking [33]. In Figure 3B, the surface hydrophobicity index of myofibrin increased in each group with the duration of heat treatment. This result may be attributed to the breakdown of the hydrophobic areas within myofibrillar proteins under elevated temperature conditions, which exposes more hydrophobic groups and, thus, increases the hydrophobicity index. Moreover, high temperatures also increased the interactions between protein molecules, resulting in the formation of larger aggregates, leading to an increase in surface hydrophobicity. This trend is in accordance with the results reported by Mitra et al. [34]. Furthermore, all experimental groups exhibited a significant decrease in the surface hydrophobicity index compared to the blank group after undergoing identical heat treatment durations (*p* < 0.05). The results suggest that shikimic acid may interact with proteins to form polymers that inhibit the unfolding of protein structures. The myofibrillar protein structure prevents the exposure of hydrophobic amino acids, resulting in a decrease in its surface hydrophobicity [35]. Compared with the group with a mass ratio of shikimic acid to myofibrillar protein of 1:50,000 (g/g), the surface hydrophobicity index of myofibrillar protein increased in all experimental groups. This may be because excess shikimic acid increases the active oxidising free radicals in the protein, resulting in an increased protein oxidation degree.

### 3.5. Endogenous Amino Acids

**Figure 4:**
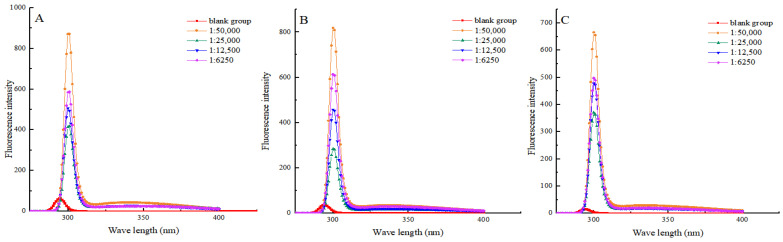
Effects of shikimic acid at 10 min (**A**), 20 min (**B**), and 30 min (**C**) of heat treatment on the endogenous amino acids of myofibrillar protein.

Molecular fluorescence spectroscopy is an analytical method used for studying the fluorescence properties of molecules. It can better reflect the oxidative deterioration of proteins and the degradation of internal amino acids [36]. The intensity of the fluorescent signal is related to the environment in which endogenous tryptophan and endogenous tyrosine reside, which, in turn, is closely related to the tertiary structure of the protein [37]. Figure 4 shows that the endogenous fluorescence intensity of myofibrillar protein decreased in all groups during the heat treatment process (10–30 min). It is shown that heat treatment may result in the disruption of protein structure, leading to changes in protein conformation and exposure of some chromophore groups containing tryptophan and tyrosine. These groups may be more inclined to cluster together rather than to interact with the surrounding water molecules, thus resulting in a decrease in fluorescence intensity. Under the same heat treatment time condition, the fluorescence intensity of myofibrillar protein in each experimental group at the emission wavelength of 300 nm was significantly enhanced compared to that of the blank group. Shikimic acid may alter the structure of myofibrillar protein, making it easier for tryptophan and tyrosine residues to interact with surrounding water molecules. This finding was consistent with the research conducted by Kumari et al. [38]. When the heat treatment time was the same, the endogenous fluorescence intensity of myofibrillar protein in the 1:50,000 (g/g) group was the highest. The results showed that excess shikimic acid induced amino acid deamination and decarboxylation, which increased the amino acid degradation rate.

### 3.6. Secondary Structure of Myofibrillar Protein

**Figure 5:**
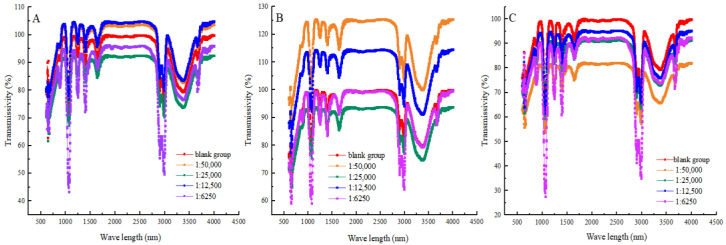
Effects of shikimic acid at 10 min (**A**), 20 min (**B**), and 30 min (**C**) on the secondary structure of myofibrillar protein.

Near-infrared spectroscopy (NIR) can measure the absorption and scattering spectra of proteins in the near-infrared spectral region. In turn, the characteristic peaks of the protein secondary structure were analysed to assess the extent of protein structural denaturation [39]. In Figure 5 and Table 2, the percentage of α-helixes, β-sheets and β-turn structures decreased, and the percentage of irregular structures increased in the blank group with the prolongation of heat treatment time. The results showed that, during heat treatment, the α-helix structure loosened and the β-sheet structure expanded or relaxed. This caused the proteins to lose their original stable structure. Then, the α-helix structure and β-sheet structure were progressively transitioned into more disordered β-turns and irregular structures [40]. Also, heat treatment might induce an expansion of the proportion of disorganised structures in proteins, making them looser and more random. Compared with the blank group, when the ratio of shikimic acid to myofibrillar protein was 1:50,000 (g/g), and the heat treatment was for 30 min, the proportion of α-helixes and β-sheets in the myofibrillar proteins increased by 16.72% and 24.74%, respectively, and the proportion of irregular structure decreased by 56.23%. This showed that moderate amounts of shikimic acid can maintain the overall stability of protein secondary structure by reducing the proportion of irregular structures. The above results were similar to those of Serson [41] and Carbas [42] et al. At an equivalent duration of heat treatment, the proportion of irregular structures increased in other experimental groups where the mass ratio of shikimic acid to protein exceeded 1:50,000 (g/g). The results showed that, when the mass ratio of shikimic acid to myofibrillar protein was 1:50,000 (g/g), the protective effect was the best. Therefore, excessive content of shikimic acid may break the chemical bonds in proteins and thus increase the degree of protein degradation.

### 3.7. Particle Size

**Figure 6:**
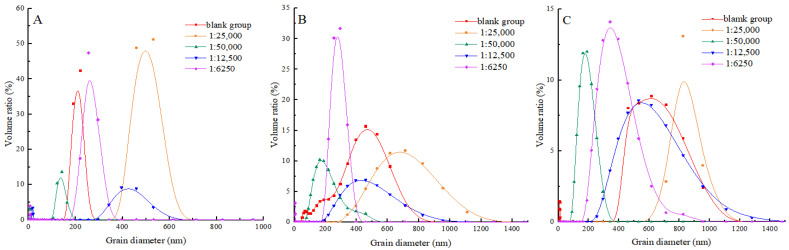
Effects of shikimic acid at 10 min (**A**), 20 min (**B**), and 30 min (**C**) on the particle size of myofibrillar protein.

During the process of heat treatment, proteins undergo cross-linking and form larger aggregates. The size of these aggregates can be characterised by the particle size of MP. It visually reflects the degree of protein crosslinking during heat treatment, thereby elucidating the effect of shikimic acid on the thermal denaturation of MP under high-temperature conditions [43]. As can be seen from Figure 6, with the prolongation of heat processing time, the protein particle size enlarged in both the blank and experimental groups. This may be because, at high temperatures, the non-covalent interactions between protein molecules are destroyed, which makes the protein molecules lose their stability, thus leading to aggregation. This aggregation increases the particle size of the protein. This was in line with the results of Ince-Coskun et al. [44]. When the mass ratio of shikimic acid to myofibrillar protein was 1:5000 (g/g), and the heating time was 10 min, the myofibrillar protein exhibited the smallest particle size and the narrowest range of particle size distributions. This indicated that shikimic acid could significantly inhibit protein aggregation and enhance protein particle size distribution, thus maintaining protein stability. When heating for 10 min, the particle size of the experimental group was larger than that of the blank group when the mass ratio of shikimic acid to myofibrillar protein was 1:6250 (g/g). It is possible that the excessive presence of shikimic acid facilitated an elevation in the content of protein disulfide bonds, consequently leading to an augmentation in the size of protein particles. A similar phenomenon was observed by Li [45] and Saleem [46] in the study of fish myofibrillar proteins and chicken actinomyosin.

## 4. Conclusions

This study employed multispectral analysis, DSC, and near-infrared spectroscopy to investigate the structural changes of duck myofibrillar protein after the addition of shikimic acid during heat treatment. These changes included a decrease in protein surface hydrophobicity, a significant increase in disulfide bonds, and a decrease in its random coil content. Shikimic acid can effectively inhibit the formation of carbonyl groups and the loss of sulfhydryl groups, as well as increase the scavenging rate of free radicals when undergoing thermal processing. These findings reveal that shikimic acid could efficiently decrease the oxidative damage to MP caused by thermal processing. The addition of shikimic acid increased the denaturation temperature and initial denaturation temperature of MP while decreasing heat absorption. The findings demonstrated that the addition of shikimic acid significantly enhanced the thermal stability of myofibrillar protein. The NIR analysis further confirmed a reduction in random structure proportion and an increase in particle size. These results suggested that shikimic acid facilitated the formation and clumping of protein structures. Therefore, the addition of shikimic acid in duck meat processing is essential for improving the stability of processed duck meat products. This study is expected to furnish scientific backing for the enhancement of duck meat product processing techniques.

## Figures and Tables

**Table 1:** Effect of the shikimic acid on the denaturation temperature and heat absorption of muscle fibrin.

Shikimic Acid to Protein Mass Ratio	Denaturation Temperature/(°C)	Heat Absorption/(J·g^−1^)	Initial Denaturation Temperature/(°C)
Blank group	69.93	1825.00	93.85
1:50,000	107.35	118.42	115.68
1:25,000	76.62	1674.00	96.04
1:12,500	106.24	125.43	114.35
1:6250	91.99	374.10	100.27

**Table 2:** Near-infrared spectroscopy parameters of unconjugated and conjugated shikimate myofibrillar protein. Note: different letters in the figure indicate significant differences (*p* < 0.05).

Peer Group	Heat Treatment Time/Min	α-Helix Ratio/%	β-Sheet Ratio/%	β-Turn Ratio/%	Irregular Structure Ratio/%
Blank group	10	12.52 ± 0.53 ^a^	47.84 ± 0.65 ^b^	29.91 ± 0.62 ^b^	9.73 ± 1.12 ^a^
	20	6.49 ± 0.22 ^a^	9.60 ± 1.20 ^a^	37.80 ± 3.71 ^b^	46.11 ± 3.27 ^c^
	30	1.95 ± 0.37 ^a^	15.73 ± 1.27 ^a^	6.81 ± 1.71 ^a^	75.51 ± 4.71 ^c^
1:50,000	10	36.53 ± 2.02 ^c^	34.45 ± 2.13 ^a^	20.50 ± 1.25 ^a^	8.52 ± 1.54 ^a^
	20	13.50 ± 1.22 ^b^	57.06 ± 1.43 ^c^	22.26 ± 1.85 ^a^	7.18 ± 1.14 ^a^
	30	18.67 ± 1.28 ^c^	40.47 ± 1.70 ^d^	21.58 ± 2.21 ^b^	19.28 ± 2.24 ^a^
1:25,000	10	17.79 ± 1.30 ^b^	30.85 ± 5.11 ^a^	20.36 ± 0.77 ^a^	31.00 ± 2.60 ^c^
	20	22.40 ± 2.64 ^c^	32.16 ± 4.18 ^b^	18.94 ± 1.49 ^a^	26.50 ± 3.54 ^b^
	30	12.57 ± 1.33 ^b^	25.09 ± 3.66 ^b^	28.43 ± 2.34 ^c^	33.91 ± 2.88 ^b^
1:12,500	10	21.29 ± 3.22 ^b^	35.73 ± 2.71 ^a^	20.37 ± 1.95 ^a^	22.61 ± 2.03 ^b^
	20	19.82 ± 2.34 ^c^	33.52 ± 4.02 ^b^	21.38 ± 1.06 ^a^	25.28 ± 2.83 ^b^
	30	15.27 ± 1.30 ^bc^	29.38 ± 3.35 ^bc^	23.30 ± 2.77 ^b^	32.05 ± 3.97 ^b^
1:6250	10	18.60 ± 2.59 ^b^	34.90 ± 1.90 ^a^	18.55 ± 1.90 ^a^	27.95 ± 2.13 ^c^
	20	21.62 ± 2.02 ^c^	30.74 ± 3.10 ^b^	19.90 ± 2.27 ^a^	27.74 ± 1.92 ^b^
	30	16.19 ± 2.51 ^c^	32.94 ± 2.12 ^c^	22.37 ± 3.65 ^b^	28.50 ± 1.90 ^b^

## Data Availability

The data presented in this study are available on request from the corresponding author. The data are not publicly available because a paper based on this part of the data is being submitted.

## Abbreviations

FTIR	Fourier transform infrared spectroscopy
ANS	8-anilino-1-naphthalene sulfonic acid
DSC	Differential scanning calorimetry
MP	Myofibrillar protein
NIR	Near-infrared spectroscopy
